# The association between retinal vein pulsation pressure and optic disc haemorrhages in glaucoma

**DOI:** 10.1371/journal.pone.0182316

**Published:** 2017-07-28

**Authors:** Dong An, Philip House, Christopher Barry, Andrew Turpin, Allison M. McKendrick, Balwantray C. Chauhan, Siobhan Manners, Stuart L. Graham, Dao-Yi Yu, William H. Morgan

**Affiliations:** 1 Center for Ophthalmology and Visual Science, Lions Eye Institute, The University of Western Australia, Perth, Australia; 2 School of Computing and Information Systems, The University of Melbourne, Melbourne, Victoria, Australia; 3 Department of Optometry and Vision Sciences, The University of Melbourne, Melbourne, Victoria, Australia; 4 Department of Ophthalmology and Visual Sciences, Dalhousie University, Halifax, Nova Scotia, Canada; 5 Faculty of Medicine and Health Sciences, Macquarie University, Sydney, NSW, Australia; Bascom Palmer Eye Institute, UNITED STATES

## Abstract

**Purpose:**

To explore the potential relationship between optic disc haemorrhage, venous pulsation pressure (VPP), ocular perfusion pressures and visual field change in glaucomatous and glaucoma suspect eyes.

**Materials and methods:**

This prospective observational study examined 155 open angle glaucoma or glaucoma suspect eyes from 78 patients over 5 years. Patients were followed with 3 monthly non-mydriatic disc photographs, 6 monthly standard automated perimetry and annual ophthalmodynamometry. The number of disc haemorrhages in each hemidisc was counted across the study period. Visual field rate of change was calculated using linear regression on the sensitivity of each location over time, then averaged for the matching hemifield. VPP and central retinal artery diastolic pressure (CRADP) were calculated from the measured ophthalmodynanometric forces (ODF). The difference between brachial artery diastolic pressure (DiastBP) and CRADP was calculated as an index of possible flow pathology along the carotid and ophthalmic arteries.

**Results:**

Mean age of the cohort was 71.9 ± 7.3 Years. 76 out of 155 eyes (49%) followed for a mean period of 64.2 months had at least 1 disc haemorrhage. 62 (81.6%) of these 76 eyes had recurrent haemorrhages, with a mean of 5.94 recurrences over 64.2 months. Using univariate analysis, rate of visual field change (P<0.0001), VPP (P = 0.0069), alternative ocular perfusion pressure (CRADP–VPP, P = 0.0036), carotid resistance index (DiastBP–CRADP, P = 0.0108) and mean brachial blood pressure (P = 0.0203) were significantly associated with the number of disc haemorrhages. Using multivariate analysis, increased baseline visual field sensitivity (P = 0.0243, coefficient = 0.0275) was significantly associated with disc haemorrhage, in conjunction with higher VPP (P = 0.0029, coefficient = 0.0631), higher mean blood pressure (P = 0.0113, coefficient = 0.0190), higher carotid resistance index (P = 0.0172, coefficient = 0.0566), and rate of visual field loss (P<0.0001, coefficient = -2.0695).

**Conclusions:**

Higher VPP was associated with disc haemorrhage and implicates the involvement of venous pathology, but the effect size is small. Additionally, a greater carotid resistance index suggests that flow pathology in the ophthalmic or carotid arteries may be associated with disc haemorrhage.

## Introduction

Optic disc haemorrhage is one of the most significant predictors of glaucoma progression [1]. The visual field and estimated retinal ganglion cell count deteriorates more quickly after an episode of disc haemorrhage, in primary open angle glaucoma across a range of intraocular pressures [1–6]. However, the pathogenesis of disc haemorrhage is unclear. Many hypotheses have been proposed, including mechanical sheering by interaction of various forces at the lamina cribrosa [7, 8], and elevated venous pressure at the optic nerve head [9]. There is evidence for reduced retinal blood flow in sectors close to disc haemorrhage [10], an association with generalized vascular disease [11] and systemic hypertension [12]. There is fluorescein angiographic evidence of delayed choroidal filling and delayed retinal circulation time in subjects with glaucoma and with disc haemorrhage [13]. This suggests that arterial and / or venous flow restriction occurs.

Spontaneous retinal vein pulsation is a phenomenon observed in most healthy eyes. In glaucomatous eyes this occurs much less frequently [14]. In eyes without spontaneous venous pulsation, an ophthalmodynanometer can be utilised to measure vein pulsation pressure (VPP) by exerting force through a handheld contact lens. Modern ophthalmodynamometers use a pressure transducer attached to the contact lens to record the force applied [15]. This ophthalmodynanometric force (ODF) is converted to VPP using published formulae [16]. Previous studies have shown that VPP is predictive of glaucoma progression [17–21]. Additionally, increased VPP appears to be indicative of elevated venous resistance within the lamina and prelaminar regions and also elevated retinal venous pressure [16].

We hypothesized that if raised venous pressure is a cause of disc haemorrhage, then we may see an elevated VPP in these patients. We explored the relationship between two glaucoma progression predictors: disc haemorrhage and VPP, along with various ocular arterial perfusion parameters derived from ophthalmodynamometric central retinal artery diastolic pressure (CRADP) measurements. The CRADP measures arterial perfusion of the eye more directly than brachial artery blood pressure (BP), and was primarily used in the analysis.

## Methods

The research followed the tenets of the Declaration of Helsinki. Ethics approval was granted by the University of Western Australia Human Ethics Committee. Glaucoma patients at two practices were recruited between 2007 and 2009 for a 5 year study with written informed consent and opportunities for questions. Consecutive patients were invited to participate when they met the inclusion and exclusion criteria. There was no age, sex and ethnicity requirements. The subjects were required to have glaucoma in at least one eye, defined as a repeatable glaucomatous visual field defect (either arcuate or nasal step pattern having at least 2 contiguous points with thresholds below the P = 0.01 level) with a positive glaucoma hemifield test (measured with Humphrey-Zeiss Analyser, Carl Zeiss Meditec, Dublin, CA) and a matching, congruent optic disc defect (focal rim notch or excavation) based on clinical opinion. The fellow eye must have had either glaucoma or be a glaucoma suspect, or with elevated intraocular pressure (IOP) more than 21 mmHg (ocular hypertension). Glaucoma suspect is defined as eye with disc rim thinning and/or excavation, but with no visual field changes as discussed above. The eyes were required to have clear ocular media for examination and photography. Subjects with both visual field mean deviations worse than -18 dB at baseline were excluded. Patients with diabetes, angle closure eyes and previous glaucoma filtration surgeries were excluded.

Central corneal thickness was measured with the Pachymate (DGH Technology, Inc. Pennsylvania, USA) at baseline. Intraocular pressure was measured with Goldmann applanation tonometry every 3 months. Brachial artery BP was measured annually using automated sphygmomanometry, with the arm elevated and the cuff at eye level in order to more closely match carotid artery pressure at eye level [22]. Use of Antiplatelet and/or anticoagulant medication was recorded.

### Follow-up

The patients were given usual care consistent with National Health and Medical Research Council Glaucoma Guidelines [23]. A step-wise escalation in therapy was given consisting of medical then selective laser trabeculoplasty then trabeculectomy with mitomycin C, when clinical progression or consistently elevated IOP was detected, with the aim of reducing IOP by 20%. Clinical visual field progression was determined by the treating clinician judging the visual field series according to their preference and using the Guided Progression Analysis event detection software. Clinical optic disc progression was determined by the clinician using the Heidelberg Retinal Tomograph Topographic Change Analysis package (Heidelberg Engineering, Heidelberg, Germany) and yearly stereo-photograph comparisons.

All patients were seen every 3 months for IOP measurement and non-mydriatic optic disc photography (CR-2 Non-mydriatic fundus camera, Canon, Tokyo, Japan). Visual field testing using the Humphrey 24–2 SITA test strategy and confocal laser scanning tomography (Heidelberg Engineering, Heidelberg, Germany) was performed every 6 months. Dilated examination with ophthalmodynamometry, VPP measurement and stereo-disc photography was performed annually.

### Disc photos

Disc haemorrhage data was collected using the series of non-mydriatic disc photographs. These photos were collated and aligned as layers using Adobe Photoshop (Adobe Systems, San Jose, CA, USA). 3 masked experts (WH.M., P.H.H., C.B.) were instructed to review the photographs using a flicker chronoscopy technique [24]. The photographs were de-identified and presented in time sequence order within each layer set. The date and location of the disc haemorrhages were recorded, with the location recorded based on a modified Garway Heath sector map of the optic disc [25], where 2 additional sectors were added by bisecting the nasal and temporal disc/field horizontally creating a total of 8 sectors. The location was also recorded as occurring in the superior or inferior hemidisc to facilitate correlation with hemivein pulsation. A confirmed disc haemorrhage was said to have occurred if two or more observers recorded a disc haemorrhage in the same photograph and location. If only one observer recorded a disc haemorrhage, the decision of a fourth observer (D.A.) was taken as the confirmation. Disc haemorrhages last approximately 6 weeks [26]. A study designed to collect all disc haemorrhages would need at least 6 weekly retinal photos, which is difficult in terms of clinic logistics. We took photographs every 3 months with the aim of capturing approximately one half of occurring haemorrhages over 5 years.

### Automated perimetry

Humphrey standard automated perimetry threshold sensitivity (dB) at each of the 54 points were recorded from each eye every 6 months. A 5-year progression slope and p-value for each point was calculated using linear regression of sensitivity versus time. Only points with progression P-value of < 0.05 were considered to have reliably changed. All other points with P-value of > 0.05 had recorded slopes of 0. Mean progression slopes for each hemi-field was calculated using the mean of all the 26 slopes, excluding the blind spot. We chose not to use regression on the mean hemifield sensitivity because of the likelihood that highly variable regions would mask the ability to detect change in other regions. If a patient was new to automated perimetry at the start of study, a learning session was provided and the result omitted. The baseline visual field was required to have less than 3 fixation losses, and less than 5% false positive and false negatives. The test was repeated if these conditions were not met. Follow up tests were used for progression analysis only (calculation of slope/rate of change) and did not have the above requirements.

### Ophthalmodynanometry

Ophthalmodynanometric force measurements were taken every 12 months for a total of 5 readings per eye. After pupil dilation and IOP measurement, the optic disc was observed with a 60 diopter condenser lens for spontaneous vein pulsation in the superior and inferior hemiveins. If present, ODF was recorded as 0 [27]. If pulsation was absent in one or both hemiveins an OcuDyn ophthalmodynanometer [27] was used to apply force to the globe until pulsation was seen and the hemivein ODF recorded. Additionally, force was increased until central retinal artery pulsation was detected indicating diastolic threshold. Three readings were recorded for each measurement and the mean was calculated. Veins that were obscured by nearby structures, i.e. artery, were excluded from analysis. Veins with non-induceable pulsations were assumed to have ODF equivalent to central retinal artery ODF. The measured ODF values were then converted to hemi-vein pulsation pressure and central retinal artery diastolic pressure (CRADP) using our previously published formula: pressure = ODF x 0.32 + IOP [27]. Brachial artery pressure was measured with the arm held so that the sphygmomanometer cuff was at eye level. We were particularly interested in the brachial artery diastolic blood pressure (DiastBP). The BP recorded in this position more accurately reflects carotid artery pressure [19].

Ocular perfusion pressure, the difference between ocular arterial and venous pressure is difficult to measure. It is likely that CRADP, rather than brachial artery pressure, is closer to ophthalmic artery pressure and better reflects the true ocular arterial pressure [22]. Traditionally ocular venous pressure was assumed to be equivalent to IOP but in subjects without spontaneous vein pulsation, the ocular venous pressure probably exceeds IOP and may be close to VPP [16]. For these reasons we have included two ocular perfusion parameters in this study: The conventional ocular perfusion pressure using CRADP–IOP (conventional OPP), and the alternative, but potentially more accurate measurement of ocular perfusion pressure using CRADP–VPP (alternative OPP) [17]. In healthy subjects, brachial artery pressure at eye level is close to internal carotid artery pressure [28]. We used DiastBP–CRADP (Carotid Resistance Index) as an index of pressure loss along the internal carotid and ophthalmic arteries.

### Statistical analysis

The number of disc haemorrhages in the superior or inferior optic disc over the 5-year period was our response variable. The explanatory variables modelled were age, sex, diagnosis (glaucoma vs glaucoma suspect), antiplatelet/anticoagulant use, central corneal thickness, mean IOP, mean BP, mean baseline hemifield sensitivity (upper field matches lower disc and vice versa), hemifield sensitivity rate of change, VPP and CRADP. With this disc haemorrhage count data fitting a Poisson distribution, a linear mixed model with Poisson distribution was utilised for data analysis. Random effects were eye (left or right) nested within patient identity, to take into account potential correlation between upper and lower optic disc regions, as well as between right and left eyes. Univariate analysis was carried out for each explanatory variable. In the multivariate model, the least significant parameter was eliminated in a stepwise pattern, until all remaining parameters had a P-value of <0.05. The compound parameters of conventional OPP, alternative OPP and carotid resistance index were entered into separate multivariate analyses, to avoid collinearity with their individual components (i.e. OPP is collinear with IOP, carotid resistance index is collinear with CRADP and mean BP).

An additional linear mixed model with same random effects was used to check correlation between VPP and visual field parameters. The response variable was VPP, explanatory variables were baseline hemifield mean sensitivity and hemifield sensitivity rate of change.

All analyses were performed using R (R Core Team (2017). R: A language and environment for statistical computing. R Foundation for Statistical Computing, Vienna, Austria. URL https://www.R-project.org/). Parameters with P-value of <0.05 were considered significant.

## Results

A total of 155 eyes from 78 patients completed the study (Table 1; data in S1 File). Due to disc haemorrhage being a count data, 4 patients with insufficient follow up time (less than 49 months) and clear disc photographs (less than 14) were excluded from data analysis. The ethnicities of patients were Caucasian (91.1%), East Asian (5.1%) and South Asian (3.8%). The mean follow up time was 64.2 months, ranging from 49.9 to 73.3 months. Each eye had a mean 20.14 ± 2.08 disc photos taken during this period. Most patients were women (70.5%) and most eyes were diagnosed with primary open angle glaucoma (63.9%). Mean IOP was 14.69 ± 2.54 mmHg which reflects a treated cohort of glaucoma patients. Average baseline field mean deviation of the cohort was -4.80 dB, with a baseline hemifield mean sensitivity of 24.57 dB. The calculated mean hemifield progression slope was -0.11 ± 0.26 dB/year.

**Table 1:** Clinical characteristics of study participants. Measurements taken over 5 year study period.

	155 eyes from 78 patients ^1^
Mean follow up time (Months)	64.19 ± 3.86 Range [49.93, 73.27]
Age	71.86 ± 7.26
Sex	F = 55 (70.5%) M = 23 (29.5%)
Glaucoma type (N = 155)	
- Glaucoma suspect/Ocular hypertension	19.3%)
- Primary open angle glaucoma	63.9%)
- Normal tension glaucoma	10.3%)
- Pigmentary	(1.9%)
- Steroid induced	(0.6%)
- Pseudoexfoliation	(2.6%)
- Juvenile	2 (1.3%)
Central corneal thickness (μm)	527.6 ± 34.1
Intraocular pressure (mmHg)	14.69 ± 2.54
Blood pressure–Systolic / Diastolic (mmHg) ^2^	117.22 ± 18.38 / 64.17 ± 8.38
Blood pressure–Mean (mmHg)^2^	81.89 ± 10.94
Visual field change (db/year)	-0.11 ± 0.26
Hemifield baseline mean sensitivity (db)	24.57 ±6.50
Global baseline mean deviation (db)	-4.80±5.02
Range of baseline mean deviation (db)	[-22.67, 2.52]
Non-mydriatic photos per eye	20.14 ± 2.08
Antiplatelet/Anticoagulant use	12 patients on aspirin. 2 patients on warfarin. (17.9%)

^1^One patient had only one eligible eye completed the study. One eye became blind during study.

^2^Brachial artery pressure measured with cuff elevated to eye level. Data are given as mean ± standard deviation whenever indicated

**Fig 1:**
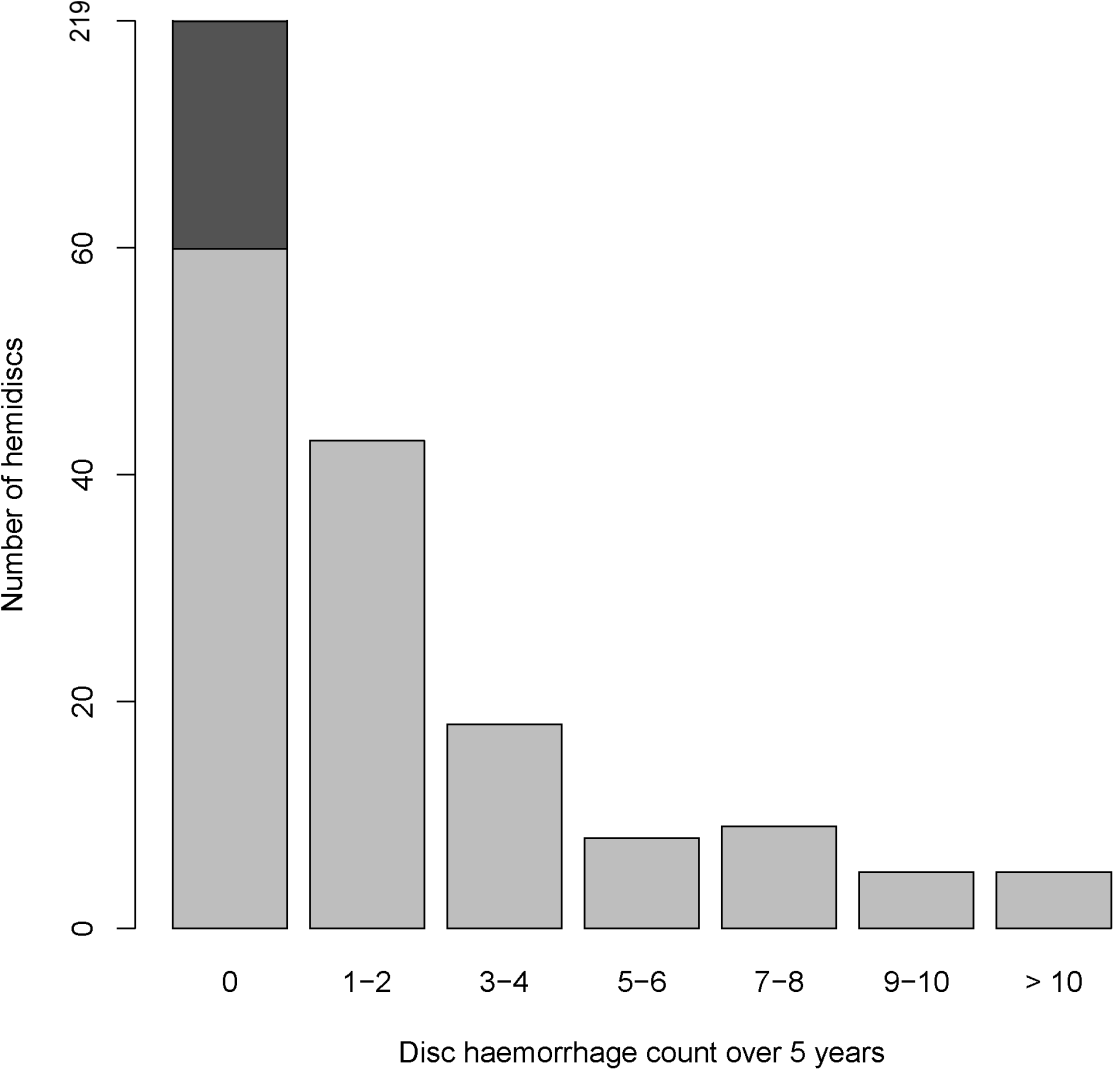
Breakdown of optic disc haemorrhage counts by hemidisc over the 5-year study period.

Mean number of detected disc haemorrhages was 2.46 per eye. 76 out of 155 eyes (49.0%) had at least one disc haemorrhage. Out of these, 62 (81.6%) had recurrent haemorrhages, with a mean of 5.94 haemorrhages over the 5-year period. This data was also presented in hemidisc format (Table 2; Fig 1), which was utilised in our regression analyses. In this study, glaucomatous eyes and glaucoma suspect eyes had similar rates of disc haemorrhage (P = 0.9647)

**Table 2:** Disc haemorrhage data over 5 year study period.

	Disc haemorrhage statistics by eye (N = 155)	Disc haemorrhage statistics by hemidisc (N = 310)	Eyes with glaucoma * (N = 125)	Eyes that are glaucoma suspects (N = 30)
With no haemorrhage	79 (51.0%)	219 (70.6%)	64 (51.2%)	15 (50.0%)
With one haemorrhage	14 (9.0%)	24 (7.7%)	12 (9.6%)	2 (6.7%)
With recurrent haemorrhage	62 (40.0%)	67 (21.6%)	49 (39.2%)	13(43.3%)
Mean no. of haemorrhages	2.46 ± 3.72	1.23 ± 2.73	2.48 ± 3.78	2.37 ± 3.46
Mean no. of haemorrhages if recurrent	5.94 ± 3.79	5.34 ±3.54	6.10 ± 3.84	5.31 ± 3.50

* Includes primary open angle, normal tension, steroid induced, pseudoexfoliation and pigmentary glaucoma.

**Fig 2:**
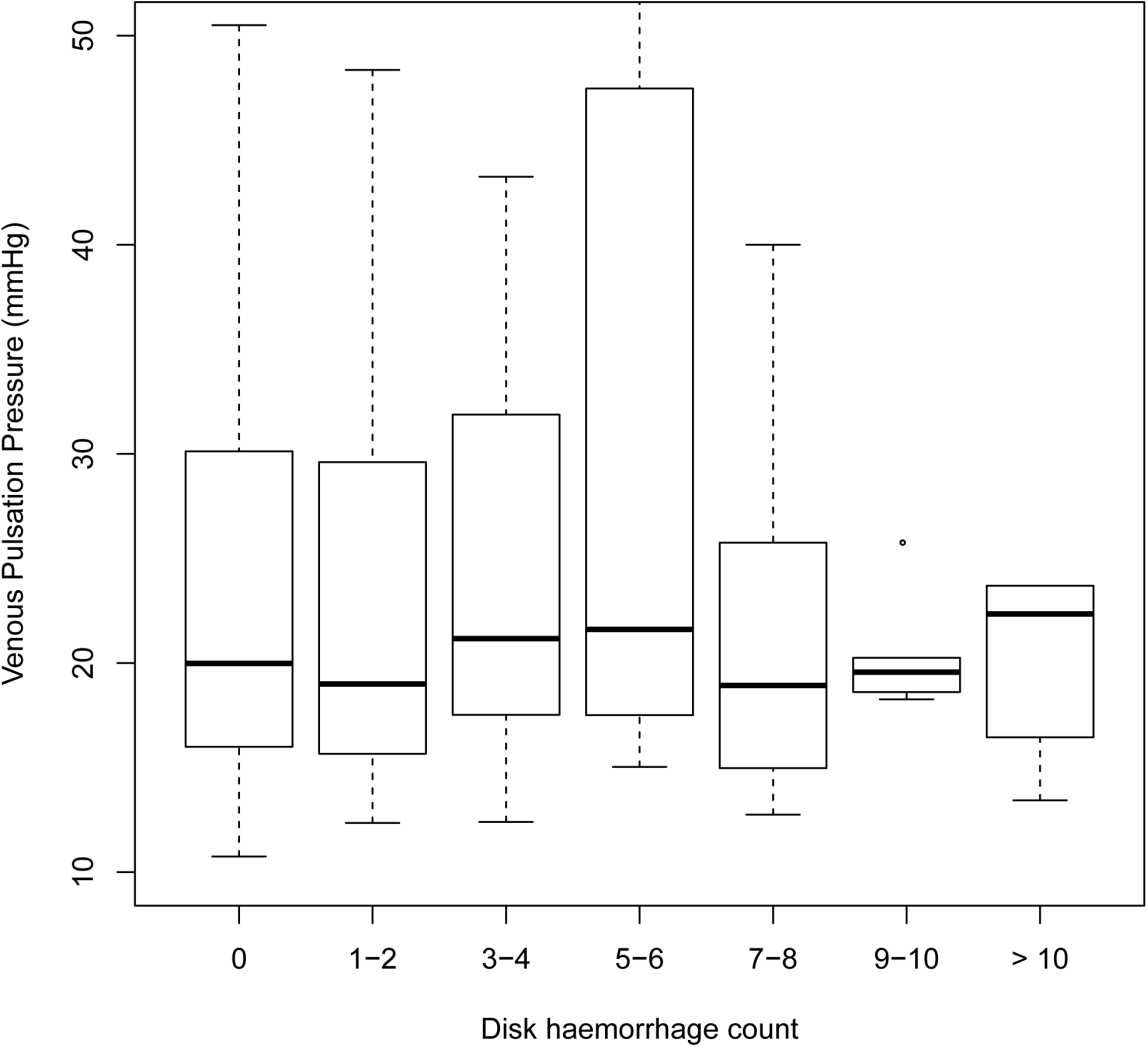
Boxplot of venous pulsation pressure (mmHg) against optic disc haemorrhage count of the corresponding hemidisc. There was a significant correlation with a coefficient of 0.0202 and P = 0.0069 (univariate analysis). Disc haemorrhage count = the total number of observed disc haemorrhage in a hemi-disc over 5-year period.

**Fig 3:**
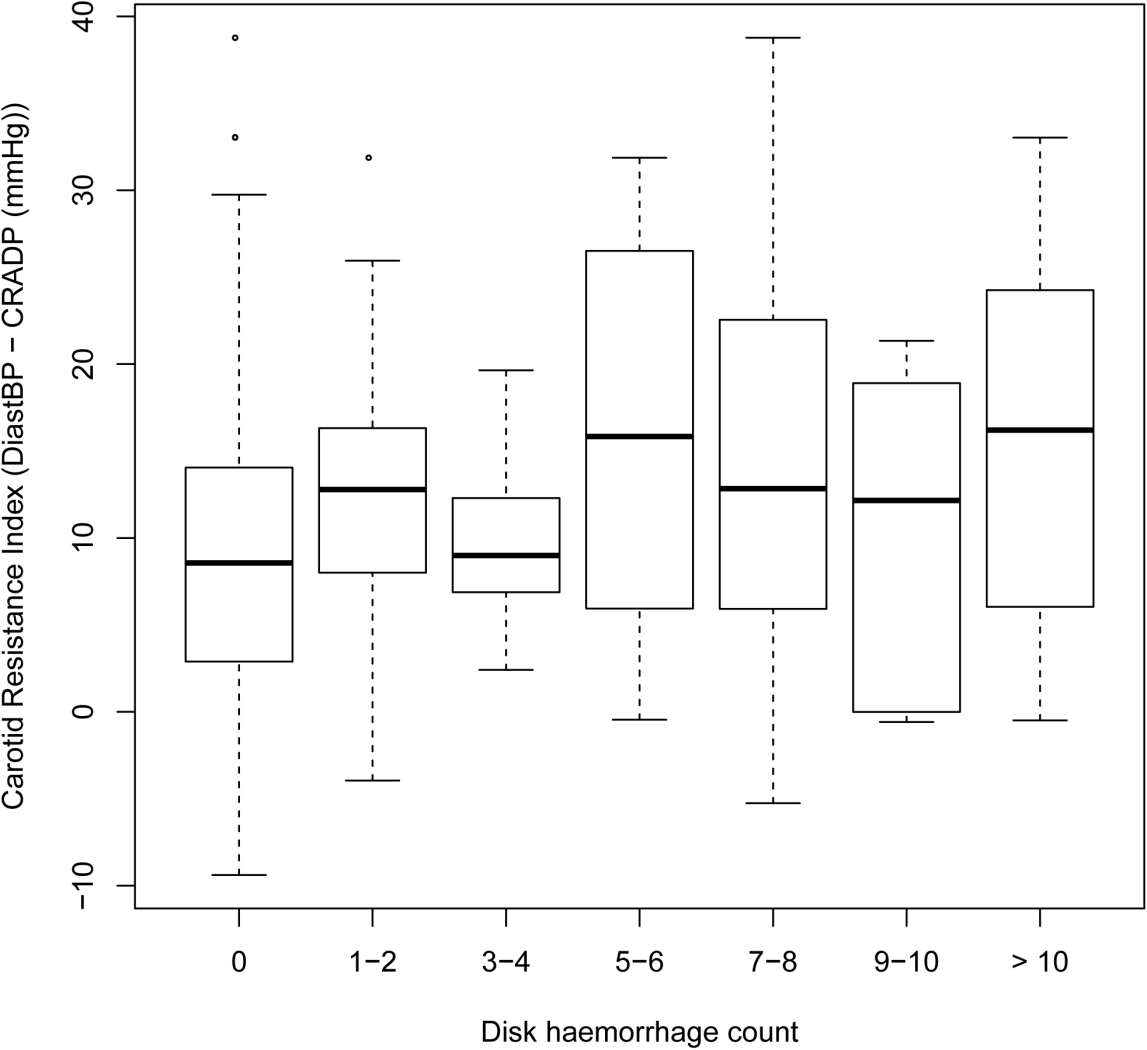
Boxplot of the carotid resistance index against optic disc haemorrhage count. There was a significant correlation with a coefficient of 0.0507 and P = 0.0108 (univariate analysis). Carotid resistance index = diastolic blood pressure measured with cuff at eye level–central retinal artery diastolic pressure.

Univariate analysis of each parameter (Table 3) revealed rate of visual field change (P <0.0001), VPP (Fig 2; P = 0.0069), alternative OPP (P = 0.0036), carotid resistance index (Fig 3; P = 0.0108) and Mean BP (P = 0.0203) were significantly associated with the number of disc haemorrhages.

**Table 3:** Univariate and multivariate linear mixed models. Association between the following parameters and optic disc haemorrhage count over 5 years. Poisson regression. Random effect = Visits|Eyes.

	Univariate analysis			Multivariate analysis		
	Coefficient	Standard Error	P-value	Coefficient	Standard Error	P-value
Age			0.3950			0.9701
Sex (male)			0.8120			0.2856
CCT			0.2540			0.2057
Mean IOP			0.4130			0.2009
**Visual field change (db/year)**	**-1.9830**	**0.2730**	**0.0000**	**-2.0695**	**0.2819**	**<0.0001**
**Hemifield baseline mean sensitivity (db)**	****	****	0.8169	**0.0275**	**0.0122**	**0.0243**
CRADP			0.4640			0.1047
**VPP**	**0.0202**	**0.0075**	**0.0069**	**0.0247**	**0.0083**	**0.0029**
Conventional OPP (CRADP–IOP)			0.6040			0.2233
**Alternative OPP** **(CRADP–VPP)**	**-0.0218**	**0.0075**	**0.0036**			0.1546
Carotid resistance index (DiastBP^1^ –CRADP)	**0.0507**	**0.0199**	**0.0108**	**0.0566**	**0.0238**	**0.0172**
**Mean BP^2^**	**0.0411**	**0.0177**	**0.0203**	0.0483	0.0190	0.0113
Diagnosis (suspect)^3^			0.5145			0.8621
Antiplatelet/ Anticoagulant			0.6440			0.7784

^1^ Brachial diastolic blood pressure measured with the cuff elevated to eye level.

^2^ Calculated using 2/3 Diastolic BP + 1/3 Systolic BP; measured with cuff elevated to eye level.

^3^ Patients grouped as 1. Glaucoma, including primary open angle glaucoma, normal tension glaucoma, steroid induced, pseudoexfoliation, and pigmentary. 2. Glaucoma suspect /Ocular Hypertension.

CCT = Central corneal thickness; IOP = Intraocular pressure; CRADP = Central retinal artery diastolic pressure; VPP = Venous pulsation pressure; DiastBP = Diastolic blood pressure; BP = Blood pressure. OPP = Ocular perfusion pressure

Multivariate linear mixed modelling using step-wise elimination of insignificant parameters revealed that VPP (P = 0.0029), carotid resistance index (P = 0.0172), mean BP (P = 0.0113) and rate of visual field change (P<0.0001) as significant predictors of disc haemorrhage (Table 3). Baseline field sensitivity (P = 0.0243) was significantly associated with disc haemorrhage in the multivariate model only.

An additional linear mixed effect analysis using VPP as the response variable and baseline field sensitivity as the explanatory variable showed strong association between the two (P = 0.0070).

The validity of the final multivariate Poisson distribution model was tested. Pearson residuals were plotted against fitted values which showed no aberrant correlations. Dispersion statistics was calculated to be 1.1611, which complies with Poisson distribution model (dispersion should be close to 1).

## Discussion

In this study with 155 glaucoma or glaucoma suspect eyes, 49% had at least one disc haemorrhage in the 5 year follow up. This is significantly higher than previously reported figures of 2–33% [29–33]. This is likely due to higher frequency of disc photos taken (disc haemorrhage has an average duration of 6 weeks) [26], flicker chronoscopy used [24], longer follow up period and having both eyes included in the study. In contrast to some previous studies, we found no correlation between disc haemorrhage and age [31, 34, 35], or sex [31, 35, 36]. Our results show that mean IOP was not a significant predictor of disc haemorrhage, which is consistent with previous studies [31, 36, 37].

Disc haemorrhage has been established as one of the most significant predictors of glaucoma progression [1, 2]. In our study, disc haemorrhage count was strongly associated with rate of field loss, with the coefficient suggesting that haemorrhage count is associated with 1.98 dB loss per year. Because haemorrhage count is the response variable, we cannot confidently state that recurrent haemorrhages leads to more field loss than a single haemorrhage. Beaufort and colleagues found that recurrent disc haemorrhages did not increase the rate of glaucoma progression in comparison to single disc haemorrhage [38]. Kim and colleague found that whilst recurrent haemorrhage caused more retinal nerve fibre layer change, the rate of visual field progression also did not differ significantly (P = 0.10) [39]. If these findings hold true for our current group of patients, our single haemorrhage coefficient of 1.98 dB per year may be underestimated in a Poisson distribution model.

There have been very few studies exploring the relationship between venous pulsation and disc haemorrhage. M. Kim et al found that spontaneous vein pulsation was not associated with disc haemorrhage (P = 0.626) [40]. Recently, K. Kim et al compared VPP in normal tension glaucoma eyes with and without disc haemorrhage [9]. Their results showed that both disc haemorrhage eyes and the fellow non disc haemorrhage eyes had lower VPP than eyes of patients with no disc haemorrhage at all. In our study, we found a small (coefficient = 0.0247; multivariate analysis) but significant positive correlation between VPP and disc haemorrhage (P = 0.0029) (Fig 2). This suggests that the effect size is quite small, which can be appreciated from Fig 2, with a VPP elevation of 49.5mmHg required to be associated with an extra disc haemorrhage. The discrepancy in our results could be due to the differences in methodology. K. Kim et al recorded the first observed central retinal vein or hemi vein pulsation as the VPP. Based on our observation and previous studies, disc haemorrhage most frequently occurs in one hemidisc location, either in the supero-temporal or infero-temporal region [29, 32]. It is a possibility that in some of their eyes, the hemivein pulsation in the non-haemorrhagic hemidisc was first observed, thus the recorded VPP may not have been co-localised to the region of the haemorrhage. The demographic differences (Korean versus mostly Caucasian) may have also contributed to the differences in our results.

Previous works consistently demonstrated the association between systemic hypertension and disc haemorrhage [12, 31, 41]. In this study, both univariate and multivariate analysis suggested that systemic blood pressure was associated with disc haemorrhage (P = 0.0113; multivariate analysis), in keeping with the results of Y.D. Kim et al and Furlanetto et al [12, 41]. The alternative OPP calculated using CRADP–VPP in our study, was significant in predicting disc haemorrhages in univariate analysis (P = 0.0036). It is not possible to say whether low perfusion pressure was associated with disc haemorrhage in our patients because this parameter was calculated from VPP, which is also indicative of venous resistance [16].

Brachial arterial pressure measured with the armband at eye level is indicative of carotid and ophthalmic artery diastolic pressure based on earlier work [27]. Thus any difference between the two is likely to be due to relative resistance change in one vessel leading to a pressure drop. The possible cause of a CRADP lower than brachial diastolic BP is narrowing along the internal carotid artery or ophthalmic artery, resulting in reduced arterial BP. The significant relationship between the carotid resistance index and disc haemorrhage count (P = 0.0172), suggests that optic nerve head hypoperfusion and ischaemia may play a role in disc haemorrhage pathogenesis. This contrasts with the significance of higher mean BP but is in keeping with overall observations by others that arterial pathology suggesting reduced perfusion pressure and flow as well as hypertension tending to increase perfusion pressure are both associated with disc haemorrhage [9–11]. In the absence of macrovascular abnormalities, higher mean BP should increase the perfusion pressure. A possible explanation is that a higher BP causes the haemorrhage to be larger, and bleed for longer, hence more likely to be detected.

Previous studies on the association between disc haemorrhage and the use of aspirin have had mixed results. Bojikian et al (post-phacoemulsification only) [42] and Soares et al reported a significant association [43]. Kim YD et al reported a borderline P-value of 0.079 [12] whilst Healey et al found no association [31]. In this study we found no significant association between disc haemorrhage and antiplatelet/anticoagulant use (P = 0.6440), however the sample size is small.

There are some limitations with our study. A major problem is clarifying whether an associated factor merely increases the size and hence visibility of the disc haemorrhage or is associated with its underlying cause. These data cannot resolve this problem for any of the explanatory variables. Elevated VPP probably reflects changes (increased resistance) in the larger hemi and central retinal veins [16] and probably not the tributary venules, so it is not the ideal measure of smaller venular resistance. However it is the only measurement of venous pressure currently available.

This is the first systematic, prospective study that has taken annual VPP measures, which would reduce the effect of measurement variability [27]. The retinal photos were taken every 3 months for 5 years, which is more frequent and over a longer time period than most other prospective studies.

In conclusion, higher VPP, carotid resistance index and rates of visual field progression were associated with optic disc haemorrhage in this study. Perfusion pressure has a complex relationship with haemorrhage. VPP is correlated with optic nerve haemorrhage but the low coefficient suggests that it requires very elevated VPP to be associated with increased number of optic disc haemorrhages. This does lend some support to the hypothesis that venous pathology is implicated in disc haemorrhage presence but the effect size is small.

## Supporting information

S1 File Dataset for statistics. 5 year clinical data used for statistical analysis.
(CSV)
